# Dual water-electricity cooperation improves economic benefits and water equality in the Lancang-Mekong River Basin

**DOI:** 10.1038/s41467-023-42009-8

**Published:** 2023-10-06

**Authors:** Bingyao Zhang, Yu Li, Chi Zhang, Chunhong Hu, Guangtao Fu, Ximing Cai

**Affiliations:** 1 School of Hydraulic Engineering, Dalian University of Technology, Dalian, Liaoning China; 2 China Institute of Water Resources and Hydropower Research, Beijing, China; 3 Centre for Water System, Faculty of Environment, Science and Economy, University of Exeter, Exeter, UK; 4 Department of Civil and Engineering, University of Illinois at Urbana-Champaign, Champaign, IL USA

**Keywords:** Water resources, Developing world, Energy supply and demand

## Abstract

Transboundary river cooperation provides an effective pathway to maintain regional security and sustainable development; however, its implementation is a pressing and prominent concern due to lack of appropriate compensation measures and effective incentive strategies. Here we develop a dual water-electricity cooperation (DWEC) framework that combines water and electricity trading to meet the often-conflicting demands of participating countries. The results from the Lancang-Mekong River Basin reveal that substantial benefits in both economic and social aspects can be achieved through coupling regional water and electricity trades. Economic benefits can be obtained by expanding cooperation space and thereby greatly improving the willingness of countries to participate in basin-wide cooperation. Electricity trading plays a key role in loss compensation for water exporters, ensuring no loss for any party and maximizing basin-wide benefits. Furthermore, the DWEC improves regional water use equality, especially in water shortage periods when there is severe competition among water users. The proposed cooperation framework provides a viable way to implement cooperation in transboundary river basins.

## Abstract (web summary)

Li and colleagues develop a dual water-electricity cooperation (DWEC) framework that combines water and electricity trading to meet the often-conflicting demands of participating countries in the Lancang-Mekong river basin. They discuss the potential of this framework for application in other transboundary river systems.

## Introduction

Transboundary river basin management is a geopolitical issue, as the countries involved are often embroiled in conflicts related to resource exploitation and water, energy, and food security^1,2^. Each country makes decisions based on its own rationality in seeking to maximize its benefits, following the hypothesis of the rational person in economics, but this leads to a ‘prisoner’s dilemma’ where collective irrationality is shown to lead to low efficiency and unsustainable resource utilization^3,4^. Transboundary cooperation is an effective approach to break out of this dilemma^4,5^; however, the effective implementation of cooperation strategies in practice is an enormous challenge, often due to lack of incentives for some parties involved.

**Fig. 1:**
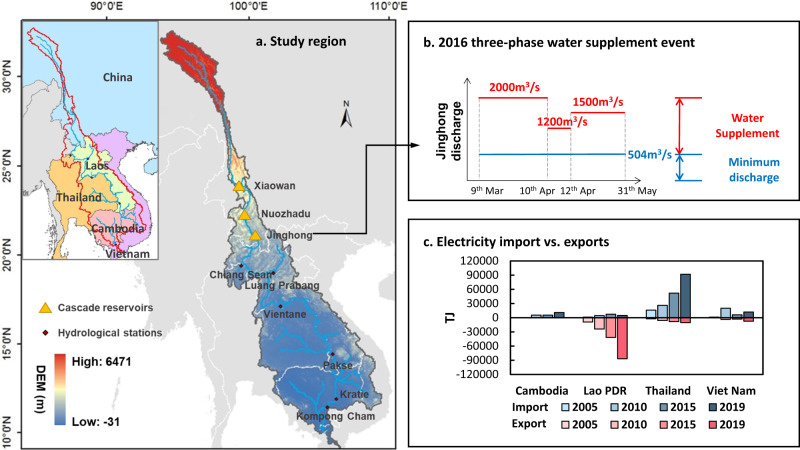
Water cooperative action and electricity trading in the Lancang-Mekong River. **a** Lancang-Mekong River Basin profile; **b** the three-phase river discharges [m^3^/s] at Jinghong (outlet of China) during the 2016 emergent water supplement initiative to alleviate the downstream drought, as requested by Vietnam; **c** electricity trade [TJ] flow of downstream Mekong countries. Data source: International Energy Agency (IEA).

The Lancang-Mekong River Basin (LMB), with a drainage area of 800,000 km^2^, supports the livelihood of ~65 million people across six Southeast Asian countries, including China, Myanmar, Thailand, Laos, Cambodia and Vietnam (Fig. 1a)^6^. Due to the natural resources variability and long-term socio-economic evolution in this area, countries in the upper basin have focused on hydropower development with cascade reservoirs, such as those constructed in China (e.g., Xiaowan and Nuozhadu) and that planned for completion by 2030 in Laos^7,8^. In contrast, agricultural irrigation is the dominant water consumption activity in downstream countries, where agricultural products contribute to a significant proportion of GDP and international trade. For example, Thailand and Vietnam are the second- and third-largest rice exporters in the world, respectively, and play an important role in the global food supply chain^9^. In recent years, population growth and frequent extreme droughts have aggravated the water scarcity problem in the basin^10,11^. To tackle this challenge, water security dialogues have been held in the LMB, with strong calls for broader cooperation^12^.

Emergency negotiation on a case-by-case basis has been the main form of cooperation in practice, and a day-to-day operational cooperation framework has not yet been established in the LMB^13,14^. One such cooperation initiative was the emergent water supplement in 2016, when the Mekong Delta was experiencing its most severe drought in the past century. During this event, the Chinese government positively responded to Vietnam’s request for emergent water supplementation (Fig. 1b), which effectively alleviated the downstream drought^15^. Emergency negotiation can provide positive impacts in the short term; however, it is unsustainable due to the following limitations. First, upstream countries with geographical advantages can feel that their benefits are being undermined and their losses going uncompensated^2^. Second, ‘free-riding’, where some countries within a coalition benefit from such cooperation without incurring any costs, is common, prompting some countries to be less motivated to participate in the coalition^14,16^. In addition, emergency negotiation focusing on water has not explored cooperation among multiple systems and sectors (e.g., the power system) to maximize social impacts and economic benefits.

Proactively incorporating the water-food-energy nexus into transboundary basin management should be explored as an opportunity to develop appropriate compensation measures and incentive strategies for all parties^6,17–19^ and may thus provide an opportunity to break the cooperation dilemma. Countries involved in the LMB have a strong appetite for electricity trading (Fig. 1c). At present, electricity is mainly exported from China and Laos to Thailand and Vietnam, while the electricity demand and supply of riparian countries are still imbalanced^20–22^, limiting the implementation of a plan that is optimal for all countries. Previous research highlights the complex nexuses among water, energy, and food systems^23^. However, transboundary cooperation is separately developed in the water and electricity sectors without addressing these nexuses.

Here we present a dual water-electricity cooperation (DWEC) framework that promotes collaborative willingness by providing a balanced distribution of costs and incentives across all parties. To our knowledge, this research is the first to couple electricity trading and water cooperation, serving as a doable and sustainable compensation and incentive measure to realize effective transboundary river basin cooperation. We assess this dual cooperation framework from both economic and social perspectives using a newly developed integrated model consisting of water and electricity modules (see “Methods”). Two types of water cooperation, i.e., emergent water supplementation (EWS) and basin-wide cooperation (BWC), are investigated, as shown in Table 1. To address the significant challenges posed by expensive long-distance transmissions and a lack of stakeholder cooperation in transboundary electricity trading, the electricity module considers two objectives: cost minimization (CMin) and willingness maximization (WMax). Specifically, willingness represents stakeholders’ preferences for electricity trade volume, with the maximum willingness achieved at the supply and demand equilibrium. More details can be found in “Methods” and Supplementary Note 3. We compare dual water-electricity cooperation with water-only cooperation scenarios (Table 1). Both economic benefits and regional equality are then quantified under these scenarios. Our quantitative results show that the dual water-electricity strategy has significant advantages in economic benefits and regional equality under all scenarios. This strategy ensures that all parties benefit from cooperation and enables a win–win framework, which would be impossible to achieve in water-only scenarios. Dual cooperation leads to a more even allocation of agricultural water uses to downstream countries and narrows the electricity demand and supply gaps for all parties. These results show the advantages of using dual water-electricity cooperation to create a practical and win–win pathway for sustainable transboundary river basin cooperation.

**Table 1:** Scenario description

Scenario		Water cooperation	Electricity trade
I	BAS	Baseline scenario, i.e., no cooperative initiative taken in the water field	Not incorporated
II	EWS	Emergent water supplement from China to downstream countries, as occurred during the 2016 drought	Not incorporated
III	EWS_CMin	The same as (II)	Cost minimization with the ‘no loss’ principle
IV	EWS_WMax	The same as (II)	Willingness maximization: seeking maximum benefits for all parties
V	BWC	Basin-wide Water Cooperation: optimizing the benefits across all parties in the entire basin	Not incorporated
VI	BWC_CMin	The same as (V)	Cost minimization with the ‘no loss’ principle
VII	BWC_WMax	The same as (V)	Willingness maximization: seeking maximum benefits for all parties

## Results

### Incorporating electricity trading into emergent water supplement increases economic benefits

We re-examine the historical cooperation initiative in 2016 in a scenario (the EWS scenario) and develop two electricity trading scenarios (EWS_CMin and EWS_WMax) for comparison. Table 1 summarizes the cooperative actions of parties in the field of water and electricity in each scenario. The baseline scenario (BAS) without cooperative initiatives is set for comparison to highlight the effectiveness of EWS. In Scenario BAS, riparian countries use water independently to meet their own demands given water availability which is mainly affected by natural geographical location. Actions in the EWS scenario are implemented in three phases (Fig. 1b): (1) from 9th March to 10th April, the average daily discharge of Jinghong Reservoir (the outlet of China, Fig. 1a) is no less than 2000 m^3^/s; (2) from 11th April to 20th April, the average daily discharge is no less than 1200 m^3^/s; and (3) from 21st April to 31st May, the average daily discharge is no less than 1500 m^3^/s. Incorporating electricity trading into the EWS scenario can ensure that no party suffers losses. This ‘no loss’ principle can be regarded as the bottom line for all parties to participate in cooperation, in line with the hypothesis of a ‘rational person’ in economics. Once this condition is met, countries may not want to further expand electricity trades to minimize the costs (EWS_CMin). Then, we further maximize the willingness of all countries with the aim of incentivizing cooperation (EWS_WMax). We assume that stakeholders’ willingness is at the highest when their electricity demand is just met by supply (with the largest net benefit), and the willingness is reduced to zero when there is no net benefit (see “Methods”).

**Fig. 2:**
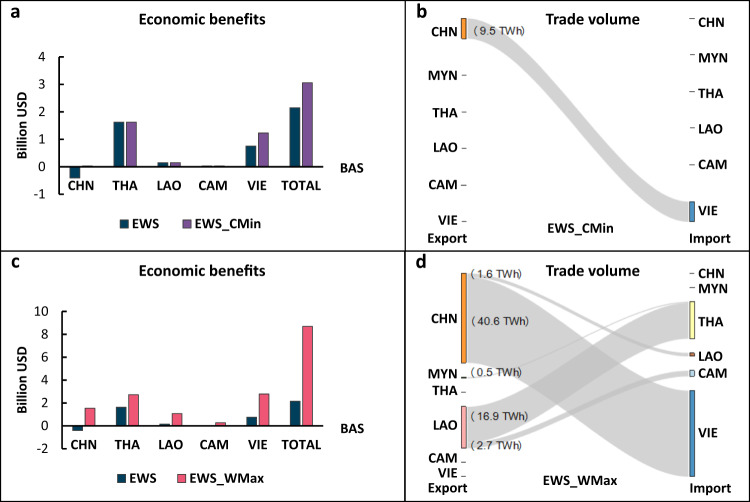
Net economic benefits and electricity trades in the 2016 emergent water supplement initiative. **a** Economic benefits from cost minimization of the coupled water-electricity system (Scenario III EWS_CMin) compared to those from the water-only Scenario II EWS. **b** Trade volume from cost minimization (Scenario III EWS_CMin). **c** Economic benefits from willingness maximization in the coupled water-electricity system (Scenario IV EWS_WMax) compared to those from the water-only Scenario II EWS. **d** Trade volume from willingness maximization (Scenario IV EWS_WMax). Note: the net benefits are calculated by the total benefits from the cooperative strategies subtracting the benefits from the BAS. Acronyms: CHN: China, THA: Thailand; LAO: Laos PDR; CAM: Cambodia; VIE: Vietnam.

The EWS scenario brought positive economic benefits to downstream countries, as seen in Fig. 2a. All downstream countries had their agricultural drought alleviated to varying degrees, leading to economic benefits, with Thailand and Vietnam experiencing the greatest benefits. However, China was deemed the only net loser.

In Scenario EWS_CMin, China has to export 9.5 TWh electricity to Vietnam to compensate for the losses incurred by the water supplement in the EWS scenario (Fig. 2b). This trade satisfies the complementary needs of China and Vietnam: China wants to export its surplus electricity, and Vietnam prefers to import electricity to meet its domestic shortfalls. Other countries (i.e., Thailand, Laos, and Cambodia) benefit from the EWS scenario, and they have no compelling need in electricity trades; thus, there are no electricity trades among them with cost-saving considerations.

In the EWS_WMax scenario, higher economic benefits are achieved for each country through more electricity trade routes and volumes (Fig. 2d). Vietnam is willing to import more electricity until its electricity gap of 40.6 TWh is met. China not only fully recovers the loss in the EWS scenario but also gains net benefits from an increased trade volume. Consequently, both countries are more willing to cooperate with each other. In addition, Myanmar exports surplus electricity to neighboring Thailand (0.5 TWh), and Laos exports surplus electricity to adjoining Thailand (16.9 TWh) and Cambodia (2.7 TWh), the expansion of electricity trade brings total economic benefits of 8.7 billion USD (Figs. 2c), 2.8 times higher than the cost minimization scenario (3 billion USD). In this sense, incorporating electricity trades in water cooperation is not only a compensation measure for upstream countries but also a means to incentivize all countries to participate in the river basin cooperation efforts.

### Basin-wide cooperation maximizes economic benefits for all countries

**Fig. 3:**
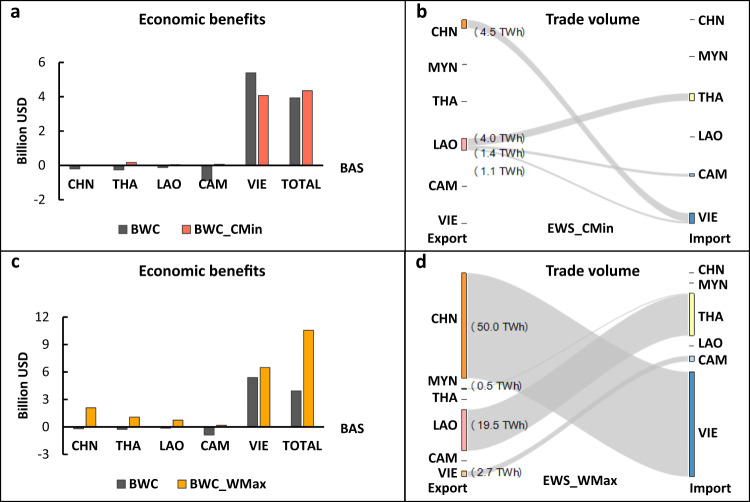
Net economic benefits and electricity trades in the basin-wide water cooperation strategy. **a** Economic benefits from cost minimization of the coupled water-electricity system (Scenario VI BWC_CMin) compared to those from Scenario V BWC. **b** Trade volume from cost minimization (Scenario VI BWC_CMin). **c** Economic benefits from willingness maximization in the coupled water-electricity system (Scenario VII BWC_WMax) compared to those from Scenario V BWC. **d** Trade volume from willingness maximization (Scenario VII BWC_WMax). Note: the net benefits are calculated by the total benefits from the cooperative strategies subtracting the benefits from the BAS. Acronyms: CHN: China, THA: Thailand; LAO: Laos PDR; CAM: Cambodia; VIE: Vietnam.

In the BWC scenario, water cooperation aims to maximize both power generation and downstream agricultural benefits by adjusting the water use processes of each party. If a basin-wide water cooperation is set up for water trade in the basin among the riparian countries, then water in the Mekong River can be temporally and spatially reallocated among riparian countries via existing river channels, with an aim to maximize the total value of water use, which depends on different crop types, crop productivity, and crops costs and prices. As water exporters, China, Thailand, Laos, and Cambodia lose water; Vietnam reaps the most benefits from water exportation (Fig. 3a). This basin-wide water transfer leads to a remarkable improvement in the total benefit of those in the water basin, i.e., 3.9 billion USD in the BWC scenario (Fig. 3a), compared to 3 billion USD in the EWS scenario.

After electricity trades are incorporated to minimize costs (BWC_CMin), the economic losses of China, Thailand, Laos and Cambodia in the BWC scenario are fully recovered (Fig. 3b): China’s loss is compensated by exporting electricity to Vietnam (4.5 TWh); Thailand and Cambodia import electricity from Laos (4.0 TWh and 1.4 TWh, respectively) to meet their electricity deficits and recover their loss; and Lao also benefits from the electricity export to Thailand, Cambodia, and Vietnam. Thus, no party incurs economic loss in the BWC_CMin (Fig. 3a) scenario, providing a sustainable means for basin-wide cooperation.

Economic benefits are further improved by expanding trade flows with willingness maximization (BWC_WMax). A higher trade flow (50.0 TWh) may appeal to China, as it seeks to export its surplus electricity (Fig. 3d), while Vietnam receives benefits from both water and electricity trades, thus increasing its willingness to cooperate. New trade routes between Myanmar and Thailand and between Vietnam and Cambodia are developed, generating more economic incentives, i.e., a total net benefit of 10.6 billion USD in BWC_WMax, which is ~2.5 times higher than the 4.3 billion USD in BWC_CMin.

Overall, the DWEC expands the cooperation potential through remarkably improved economic benefits in both the EWS and BWC cooperative strategies. Electricity trading serves as compensation for countries with water supply losses and as an incentive to promote all parties’ willingness to participate in cooperation. The DWEC provides a feasible and sustainable pathway for the implementation of transboundary river basin cooperation.

### Dual water-energy cooperation improves regional water use equality

In the process of pursuing economic benefits, resource reallocation and transfer in the transboundary river basin should address social issues, such as regional equality^24,25^. Here, we interpret the DWEC strategy from an equality perspective. As electricity resources are transferred from the surplus side to the deficit side through trade, the demand and supply of electricity resources in each country are gradually balanced (Fig. S3), which has a positive impact on regional electricity resource utilization. The reallocation of water will affect the original water uses both temporally and spatially, which can further affect water equality among the riparian countries. Here we relate water equality to the even distribution of the unit agricultural water abstraction from the river according to their planting areas across riparian countries, and quantify it using the Gini index. A higher Gini index means that water equality exacerbates as water transfers lead to a larger gap in water abstraction from the river per planting area among riparian countries.

**Fig. 4:**
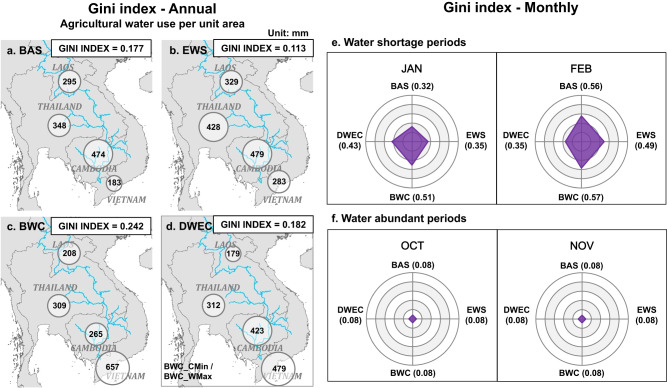
Impacts of different cooperative strategies on water use equality. **a**–**d** Agricultural water abstraction from the river per unit area in downstream countries (mm) and corresponding Gini index at the annual scale; **e** Gini index of water shortage periods; and **f** water abundant periods at the monthly scale.

In water-only cooperation, the EWS scenario improves the equality of agricultural water use among downstream countries, characterized by a lower Gini index of 0.113 (Fig. 4b) compared to the value of 0.177 achieved without cooperative action (Fig. 4a). Upstream water supplementation alleviates the extent of the water deficit of each country and mitigates the water use competition among them, thus leading to improvements in water use equality. However, the BWC scenario exacerbates the inequality of water use among downstream countries, with the Gini index increasing to 0.242 (Fig. 4c). In the pursuit of greater economic benefits, the BWC scenario allocates water in terms of unit water benefits. Vietnam, located in an estuary, can receive more water from other countries due to its relatively higher unit water benefit, and its agricultural water consumption per unit area reaches 657 mm, which is far higher than the value of 283 mm in EWS. This mainly comes at the cost of water loss in Cambodia (from 474 to 265 mm). Basin-wide water transfer changes the inherent rule that upstream countries have priority in water withdrawal and worsens the water use equality among different countries.

Incorporating electricity trades dramatically changes the spatial pattern of agricultural water use (Fig. 4d). Water is still transferred from Thailand, Laos, and Cambodia to Vietnam. However, the amount of water transfer that can be afforded by countries changes following the ‘no loss’ principle to create a win–win situation. The extent of water transfer undertaken by each stakeholder should be matched with its electricity trade capacity, which is restricted by its resource endowments and its balance between electricity supply and demand. A country can compensate its water use loss by electricity trade gain if the country has a large electricity generation capacity (e.g., China). In the DWEC, Cambodia, with its lower electricity trade capacity, has lower water losses, and its water use rises from 265 mm in the water-only cooperation BWC scenario to 423 mm; similarly, Laos, which gains more benefits in electricity trading, can afford greater water losses, and its water use decreases from 208 mm in the water-only cooperation BWC scenario to 179 mm (Fig. 4c, d). The Gini index of BWC_CMin (or BWC_WMax) is 0.182, which is lower than that in water-only cooperation BWC scenario (0.242) and therefore shows more equitable water reallocation from a social perspective.

Water use conflicts among different countries are more significant in dry periods; thus, we further assess water use equality in dry and wet periods. Water transfer exacerbates water use equality in January and February, which are the driest periods of the year (more details on the water transfer process are shown in Fig. S2). The Gini index in this period is far higher than the overall annual value, especially in February, when it reaches 0.5 in the water-only cooperation BWC scenario (Fig. 4e), which is higher than the internationally recognized ‘warning line’ of 0.4. Water use equality worsens due to the unconstrained water transfer in water-only cooperation, which shows the trade-off between economic benefits and equality. Most importantly, this finding reveals that it is inappropriate to pursue economic benefits only through water allocation, which may cause excessive water transfer from water-scarce countries, and this could be one of the factors hindering the practical implementation of water-only basin-wide cooperation. In the DWEC, the water transfer is constrained by electricity trade capacity, and water use equality improves, decreasing from 0.5 to 0.35. It should be noted that the Gini index is very low in abundant water periods (Fig. 4f) because nearly all stakeholders’ water demands can be met without water transfer. Thus, the water inequality issue may be underestimated using an annual-scale Gini index, so cooperation strategies should focus more on improving inequality periods of water shortages.

## Discussion

We show that transboundary cooperation in a novel form, i.e., dual water-electricity cooperation, can be a sustainable solution in the Lancang-Mekong River Basin based on the desires of the various parties. The DWEC is a cooperation framework that facilitates complementarity and synergy among the parties and encourages all riparian countries to participate in the cooperation initiative. The DWEC strategy shows significant improvements in both economic benefits and regional water use equality for all parties.

**Fig. 5:**
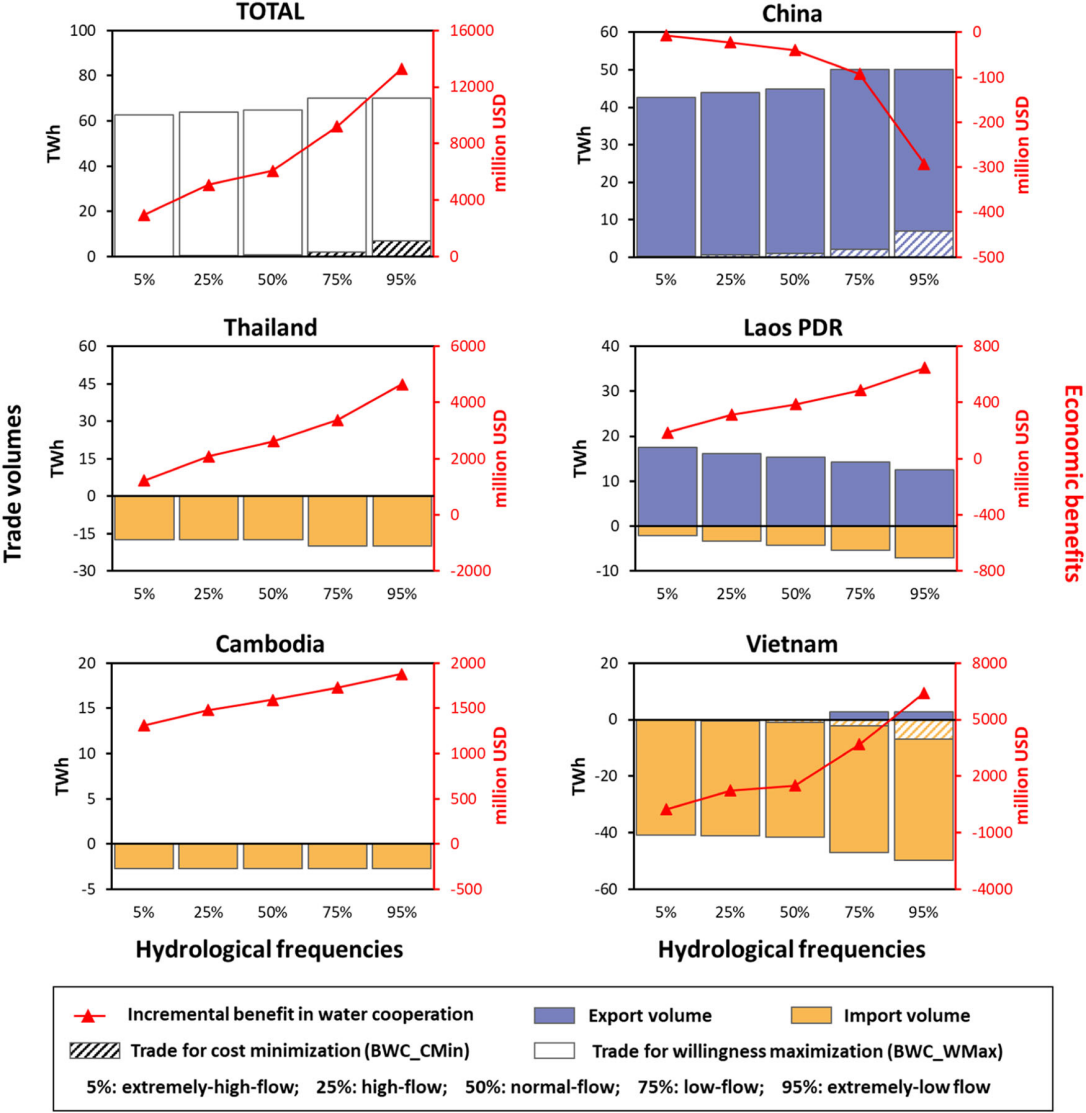
Economic benefits and electricity trades under different hydrological conditions. The red line represents the increase in economic benefits from water cooperation, and the values are given on the right-hand y-axis; the blue bar represents electricity export volumes, the pink bar represents electricity import volumes, and the values are given on the left-hand y-axis.

The cooperation effectiveness can be affected by hydrological conditions^16,26^, so we assess the DWEC strategy (Scenario V: BWC_CMin and Scenario VI: BWC_WMax) under different hydrological conditions, covering extremely high-flow conditions with a 5% hydrological frequency to extremely low-flow conditions with a 95% hydrological frequency (Fig. 5). Larger water benefits from cooperation can be obtained during severe droughts, while the total benefit improvement comes at a cost only for upstream China. As inflow decreases, China loses more water use benefits when cooperating with downstream countries; as a consequence, higher electricity trade volumes are needed for the loss compensation. However, it is noted that the electricity trade for compensation accounts for only a small proportion of the total electricity trade, the major part of the electricity trade is determined by the demands of the participants and is hardly affected by hydrological uncertainty. As a result, the total electricity trade is not significantly affected by hydrological conditions. Compared with water-only cooperation, which tends to occur in some extreme drought events, the DWEC is robust with hydrological conditions.

The socio-economic development across the basin, the growing cooperation demand, and institutional establishment for international cooperation provide potential support for the implementation of DWEC. Hydropower resources in southwest China are abundant, clean and relatively low-priced; while developing countries in Southeast Asia face large electricity shortages and high electricity prices. Thus, electricity trade could be beneficial for both sides. Actually, a report jointly published by ASEAN Centre for Energy and China Renewable Energy Engineering Institute has highlighted the benefit of transboundary electricity transmission projects^27^. Moreover, the long-term stable political environment, widespread cultural exchanges, and shared goals of regional security and sustainable development will provide political support for the proposed DWEC.

In recent decades, riparian countries have transitioned from initial cooperation to in-depth cooperation, and the Lancang-Mekong cooperation mechanism first covering all riparian countries is proposed in 2014. As of today, the first 5-year plan of Lancang-Mekong cooperation (2018–2022) has been completed, and several institutions have been established, such as Lancang-Mekong Environmental Cooperation Center, Lancang-Mekong Water Resources Cooperation Information Sharing Platform and Global Center for Mekong Studies. In August 2021, the Association of Southeast Asian Nations, Mekong River Commission (ASEAN-MRC) urged all parties to commit to deeper engagement and cooperation to boost the sustainable management of water and related resources throughout the region^28,29^. Water security and electricity interconnection are the key priorities under several cooperation frameworks, including those of the ASEAN-MRC and Lancang-Mekong. These multiparty agreements and stable political environment provide a foundation for the implementation of dual water-electricity cooperation.

However, we have to notice the challenges in the implementation of the proposed DWEC, including energy market uncertainties, transaction costs, data sharing and privacy, and the lack of legal and regulatory frameworks^27,30^. Thus, the DWEC should be further evaluated within a broader international political and economic framework. Moreover, potential environmental impacts resulting from changed hydropower reservoir operation may cause transboundary concerns, for example, the impact on sediment flow to the downstream^7,31,32^. Possible trade-off between water use and environment integrity across the riparian countries needs additional research in the context of transboundary cooperation in the Mekong River Basin.

The DWEC presents a promising and practical approach for transboundary river basin cooperation, and it can potentially be applied in other river basins. For example, with the construction and operation of the upstream Grand Ethiopian Renaissance Dam and downstream High Aswan Dam, riparian countries in the Nile River Basin have appetites in both water share and regional electricity interconnection^5,33–35^. In addition, the lack of compensation and the existence of ‘free-riding’ lead to reluctance to cooperate and even conflicts of interest. Dual water-electricity cooperation makes efficient use of limited water resources and balances regional electricity resources, helping achieve the United Nations’ Sustainable Development Goals.

## Methods

### Conceptual representation of the Lancang-Mekong River Basin

**Fig. 6:**
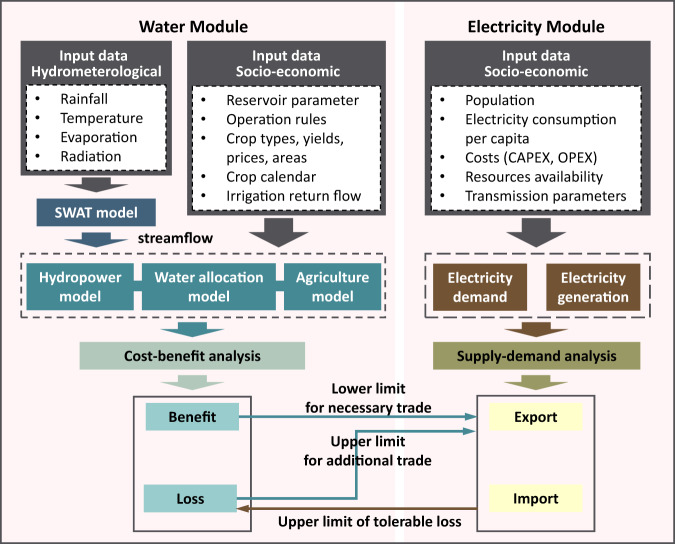
The integrated water-electricity system model. Input data, sub-models, and linkages in water module and electricity module are included.

The river flow and electricity trade relationships among riparian countries are depicted in Fig. S1. For water flow simulation, the rivers are represented by links, and key reservoirs (e.g., mega-reservoirs Xiaowan and Nuozhadu and the outlet reservoir of China: Jinghong) and hydrological stations (including Chiang Sean, Luang Prabang, Vientiane, Pakse, Kratie, and Kompong Cham) are represented by nodes. The relationships among water users are represented according to the interconnection of their geographical locations and water use strategies (Fig. S1a). For electricity system simulation, trade routes are generalized based on existing routes at the current level^27^ (Fig. S1b). China and Laos are the main exporters, Thailand and Cambodia are the main importers, and Myanmar and Vietnam usually fulfill both exporter and importer roles. It should be noted that the existing electricity trade routes directly or indirectly connect almost all countries in the basin. Therefore, we assume that the trade routes are fixed, and new trades are mainly made to expand trade capacity to meet all parties’ demands. Based on these results, we develop an integrated water-electricity system model (Fig. 6).

### Water module

The water module integrates several sub-models, which include the SWAT model, hydropower generation model, agriculture model, water allocation model, and cost–benefit analysis (Fig. 6). The hydropower generation model describes the cascade reservoir operation strategies. The agriculture model describes the process of crop growth and water requirements in different growth periods. The water allocation model is used to link different water users. Cost–benefit analysis is adopted to quantify the gains and losses of various stakeholders under different water use strategies. When there is no cooperation, the order of water use goes from upstream to downstream. When different stakeholders cooperate with each other, their water use strategies are optimized to maximize the total water benefits, including hydropower benefits and agricultural benefits. This module can accurately reproduce the 2016 historical event—the three-phase water emergent supplement initiative. Economic benefits from the water system consist of hydropower benefits and agricultural benefits.

The hydropower benefit (${BH}$) is estimated by hydropower generation and is subject to water balance, storage, turbine discharge, and capacity constraints as follows:

1 $${{BH}}_{n}={p}_{e}\mathop{\sum}\limits_{t}K\times {q}_{n,t}\times {h}_{n,t}\times \Delta t\times {\alpha }_{1}$$

2 $$s.t.\left\{\begin{array}{c}{S}_{n,t+1}={S}_{n,t}+({{in}}_{n,t}+{r}_{n-1,t}-{r}_{n})\times \Delta t\\ {\underline{S_{n}}}\le {S}_{n,t}\le {\overline{S_{n}}}\\ {\underline{q_{n}}}\le {q}_{n,t}\le {\overline{q_{n}}}\\ {\underline{nm_{n}}}\le {{nm}}_{n,t}\le {\overline{nm_{n}}}\end{array}\right.$$

where $\,n$ is the reservoir, including Xiaowan, Nuozhadu, and Jinghong; $t$ is the period, which equals 10 days; ${p}_{e}$ is the electricity price, the value of which adopted in this study is 0.05^32^ [USD per KWh]; $K$ is the efficiency coefficient, the value of which is 8.5 for large reservoirs; ${q}_{n,t}$ is the flow through turbine [m^3^/s]; ${h}_{n,t}$ is the net water head, i.e., the difference between the reservoir water level and the tailwater level of turbines [m]. The reservoir water level is obtained via the interpolation of the water level-storage curve; similarly, the tailwater level is obtained via the interpolation of the discharge-tailwater level curve. These variables change with reservoir inflow and storage; ${{in}}_{n,t}$ is the inflow for reservoir [m^3^/s]; ${r}_{n,t}$ is the total water release, including flow through turbines and spill [m^3^/s]; ${S}_{n,t}$ is the storage of reservoir [m^3^]; ${\underline{S_{n}}}$ and ${\overline{S_{n}}}$, respectively, represent the minimum and maximum storage [m^3^]; ${\underline{q_{n}}}$ and ${\overline{q_{n}}}$, respectively, represent the minimum and maximum flow through the turbine [m^3^/s]; ${{nm}}_{n,t}$ is the hydropower capacity [KW]; ${\underline{nm_{n}}}$ and ${\overline{nm_{n}}}$ represent the firm capacity and installed capacity, respectively [KW]; $\Delta t$ is the time step [s]; and ${{{{{{\rm{\alpha }}}}}}}_{1}$ is the conversion coefficient [dimensionless].

For agricultural benefits (${BA}$), the widely applied water production function proposed by the Food and Agriculture Organization of the United Nations (FAO) and farmland water balance is used to quantify these benefits. Six typical crops are selected in each country, which cover nearly 90% of the total harvest area, i.e., Thailand: rice (50%), rubber (15%), sugarcane (7%), cassava (6%), maize (5%) and oil palm fruit (4%); Laos: rice (56%), maize (12%), vegetables (10%), coffee (5%), cassava (4%), and sugarcane (2%); Cambodia: rice (74%), cassava (10%), maize (4%), soybeans (3%), vegetables (2%), and beans (2%); and Vietnam: rice (55%), maize (8%), vegetables (6%), rubber (5%), coffee (4%), and cassava (4%). The percentage is the proportion of the total planting area. Crop area, yields, and prices are given at the basin’s current level (2017). The agricultural benefit of country $m$ is estimated as follows:

3 $${{BA}}_{m}=\mathop{\sum}\limits_{k}{{RY}}_{m,k}\times {{ym}}_{m,k}\times {A}_{m,k}\times {{pr}}_{m,k}\times {\alpha }_{2}$$

where $\,m$ is the downstream country; $k$ is the type of crop; ${{RY}}_{m,k}$ represents the relative yields of crop [dimensionless]; ${{ym}}_{m,k}$ represents the potential yield [tonnes/ha]; ${A}_{m,k}$ is the planting area [ha]; ${{pr}}_{m,k}$ is the crop price [USD/tonnes]; and ${{{{{{\rm{\alpha }}}}}}}_{2}$ is the unit conversion coefficient [dimensionless].

Moreover, some environmental flow requirements are considered as constraints in the water module. The flow at the outlet of upstream China (Jinghong reservoir) is 504 m^3^/s, which is based on negotiation between China and downstream riparian countries and could guarantee navigation and basic water requirement in dry periods. Also, the minimum flows during the pre-dam period recorded by the mainstream hydrological stations are selected as the lower limit of river flow, which is to maintain the basin aquatic environment of the river.

### Electricity module

The electricity deficit or surplus is calculated as the difference between the electricity production capacity and stakeholder demand, and these are also the constraints in the electricity trade (Fig. 6). Cost is always a key factor in electricity system planning^36^; stakeholders’ willingness to participate is crucial for transboundary river basin cooperation. The above two objectives are used to measure the effectiveness of electricity trades.

We assume that no additional power plants are built for expanding cross-border electricity trading, and new trades are based on the existing infrastructure to better match spatial electricity demands. With this assumption, we focus on the cross-border transmission cost (TC) and operational expenditure (OPEX) brought by trade volume change without considering the capital expenditure of the original power plants, as follows:

4 $$\min ({TC}+{OPEX})$$

5 $${{{{{\rm{s}}}}}}.{{{{{\rm{t}}}}}}.\left\{\begin{array}{c}{TC}={\sum} _{i,\, j}{{tc}}_{i,\, j}\times {T}_{i,\, j}\times \tau /(1-{{tl}}_{i,\, j})\\ {OPEX}={\sum} _{i,\, j}{{opex}}_{i,\, j}\times {T}_{i,j}\\ {T}_{i,\, j}\le \mathop{\min }\limits_{i,j}\left\{\left|{G}_{i}-{D}_{i}\right|,\left|{G}_{j}-{D}_{j}\right|\right\}\end{array}\right.$$

where $i,j$ represent different stakeholders; ${{tc}}_{i,j}$ is the unit transmission cost of the trade flow between stakeholder $i$ and stakeholder $j$ [USD/MWh]; ${T}_{i,j}$ is the newly added trade volume between stakeholder $i$ and stakeholder $j$ [MW]; $\tau$ is the interval time, taken as 8760 h a year here; ${{tl}}_{i,j}$ is the transmission loss [%]; ${{opex}}_{i,j}$ is the unit operational expenditure of the trade flow between stakeholder $i$ and stakeholder $j$ [USD/MWh]; ${G}_{i}$ is the electricity generation of stakeholder $i$ [MW], which refers to the International Energy Agency (IEA) and the literature; and ${D}_{i}$ is the electricity demand of stakeholder $i$ [MW]. We assume that the demand is related to the population and electricity consumption per capita of each country.

**Fig. 7:**
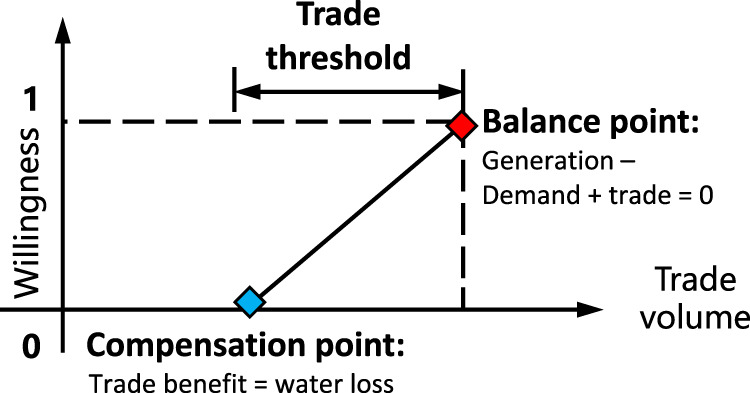
Utility functions for calculating willingness. The function shows the relationship between trade volume and willingness for participating in basin-wide cooperation.

The willingness function is characterized by a triangular utility function, showing the relationship between the stakeholder’s willingness and trade volume, which can also be regarded as a fuzzy membership function. Willingness has a maximum value of 1.0 when the supply fully meets the stakeholder’s demand (the red point in Fig. 7), and willingness takes a minimum value of 0 when the stakeholder’s benefit gain from electricity trade compensates only for water loss (the blue point in Fig. 7). According to the role of stakeholders in water cooperation (loss or gain) and electricity trade (exporter or importer), willingness can be generalized into four types; more details can be found in Supplementary Information 1.

### Linkage between water and electricity modules

Our modeling approach (Fig. 6) links two modules of water cooperation and electricity trade to develop the strategies of dual water-electricity cooperation in the LMB. To our knowledge, this is the first time that water cooperation and electricity trade have been considered in an integrated framework.

From the water module to the electricity module, the electricity trade volumes between different stakeholders are constrained by their gain or loss in water cooperation as follows:

If the stakeholder experiences losses through water cooperation, with a negative water benefit, it needs to expand its electricity trade to recover these losses. In this case, the water benefit loss is the lower limit of the electricity trade.

If the stakeholder benefits from water cooperation, with a positive water benefit, it can sustain additional trade beyond its original electricity trade demand. The losses brought by the additional trade should not exceed its water benefit gain. In this case, the water benefit gain is the upper limit of the electricity trade.

In the electricity module to the water module, the compensatory capacity of electricity trade depends on the resource endowment and demand of each stakeholder and in turn restricts water cooperation. All stakeholders need to ensure that their losses in water cooperation are within the scope of their electricity trade compensation capacity, as follows:

If the stakeholder experiences losses through water cooperation and is an electricity importer, the benefit from the electricity trade is subject to the stricter constraints of its own electricity deficit and the export electricity provided by surrounding countries. In this case, the electricity import benefit is the upper limit of the water loss that can be tolerated.

If the stakeholder experiences losses through water cooperation and is an electricity exporter, the benefit from electricity trade is subject to the stricter constraints of its own electricity surplus and the electricity import appeals of surrounding countries. In this case, the electricity export benefit is the upper limit of the water loss that can be tolerated

The water-electricity integrated model is coded in GAMS, and more details can be found in Supplementary Information 2.

### Regional equality

The essence of cooperation is the spatial and temporal reallocation of water. The spatial distribution under different cooperation strategies leads to the issue of water use inequality among various stakeholders, and we evaluate water use inequality with the intuitively described Lorentz curve and the quantitatively characterized Gini index^24,25^. The Lorentz curve is a cumulative distribution curve characterized by the proportion of planting area and the corresponding proportion of water withdrawal. The Gini index is formulated as follows:

6 $${{{{{\rm{Gini\; index}}}}}}=\frac{{\sum }_{i=1}^{m}{\sum }_{j=1}^{m}\left|{x}_{i}-{x}_{j}\right|}{2{m}^{2}\bar{x}}$$

where $x$ is the examined variable, for which we chose the agricultural water withdrawal per unit of planting area, and $m$ is the total number of agricultural stakeholders, i.e., downstream countries. A Gini index of 0 corresponds to the diagonal line of the Lorentz curve and conforms to the social justice principle of strict egalitarianism, which represents absolutely fair resource distribution. Conversely, a Gini index of 1 indicates that all resources are concentrated in a single individual and represents absolutely unfair resource distribution. In general, the Gini index ranges between 0 and 1, and the larger the value is, the more unfair the water use situation.

### Supplementary information

Supplementary Information

## Data Availability

The long-series meteorological and hydrological data are open-sources and available from various international institutions, including NASA (rainfall), ECMWF (temperature), Meteonorm (radiation), and MRC (discharge). The socio-economic data are available from reports, datasets, publications by international institutions, including Food and Agriculture Organization (FAO), International Rice Research Institute (IRRI), International Energy Agency (IEA). More details of data sources can be seen in Supplementary Note 1. Other relevant data in this research are available from the corresponding author upon reasonable request.

## Code availability

All codes are implemented via GAMS, and should be available from the corresponding author upon reasonable request.
