# Supplementary material for: Dual water-electricity cooperation improves economic benefits and water equality in the Lancang-Mekong River Basin
Source: Nat Commun. 2023 Oct 6;14:6228. doi: 10.1038/s41467-023-42009-8 (PMC10558469; doi:10.1038/s41467-023-42009-8)
Supplement: Supplementary file 1 — Supplementary Information [file 41467_2023_42009_MOESM1_ESM.pdf]

Supplementary Information for:

Dual water-electricity cooperation improves economic benefits and water equality in the Lancang-Mekong River Basin

Bingyao Zhang[1], Yu Li[1*], Chi Zhang[1*], Chunhong Hu[2], Guangtao Fu[3], Ximing Cai[4]

[1]School of Hydraulic Engineering, Dalian University of Technology, Dalian, Liaoning, China.

[2]China Institute of Water Resources and Hydropower Research, Beijing, China.

[3] Centre for Water System, Faculty of Environment, Science and Economy, University of Exeter, Exeter, UK.

[4]Department of Civil and Engineering, University of Illinois at Urbana-Champaign, Champaign, IL, USA.

*Correspondence to: liyu@dlut.edu.cn; czhang@dlut.edu.cn .

Contents:

Supplementary Figure 1: Topological map of relations among countries.

Supplementary Figure 2: Water deficit in various countries under different strategies.

Supplementary Figure 3: Electricity deficit or surplus and trade power in dual water-electricity cooperation.

Supplementary Figure 4: Runoff simulation results of the SWAT model

Supplementary Table 1: Data sources

Supplementary Table 2: The calibration and validation period of the SWAT model

Supplementary Note 1: Data sources

Supplementary Note 2: Calibration and validation of the SWAT model
Supplementary Note 3: Willingness function.
Supplementary Note 4: Linkage between water and electricity module.

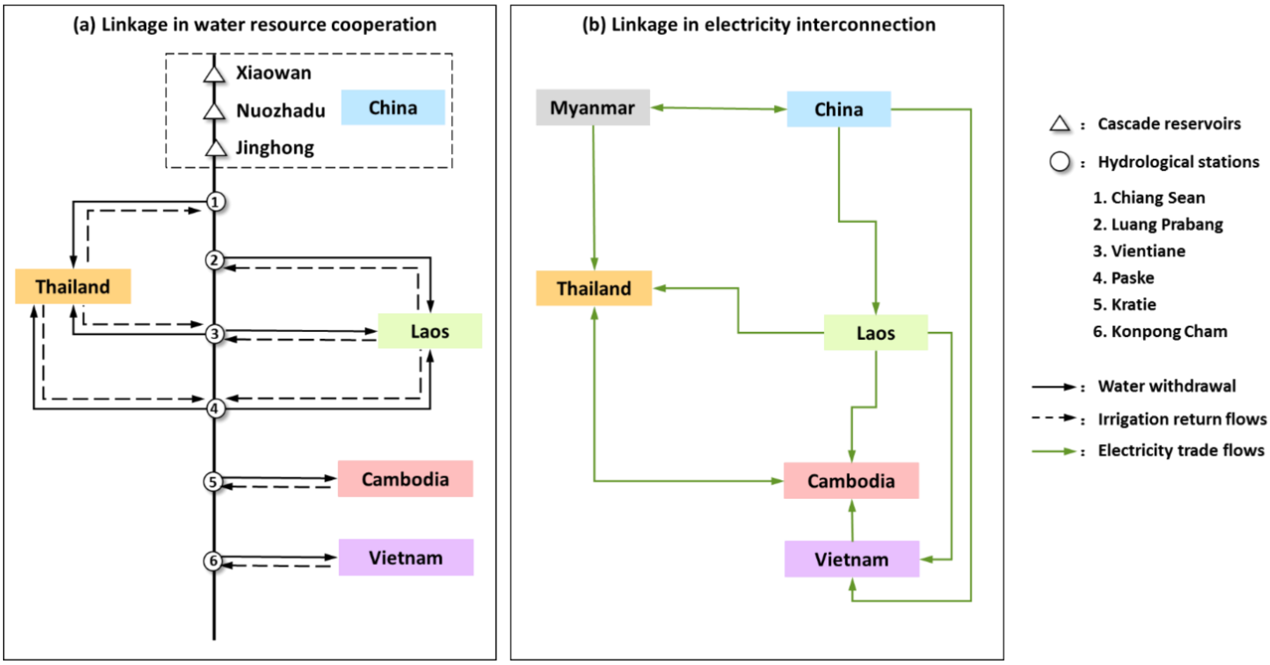

Supplementary Figure 1: Topological map of relations among countries. a. Linkage in water resources cooperation; b. Linkage in electricity interconnection.

Note: Myanmar is not considered in the water cooperation because it only accounts for 3% of the basin area and almost does not take water from the Mekong River; its geographical location is important in the electricity trade which connected China and Thailand.

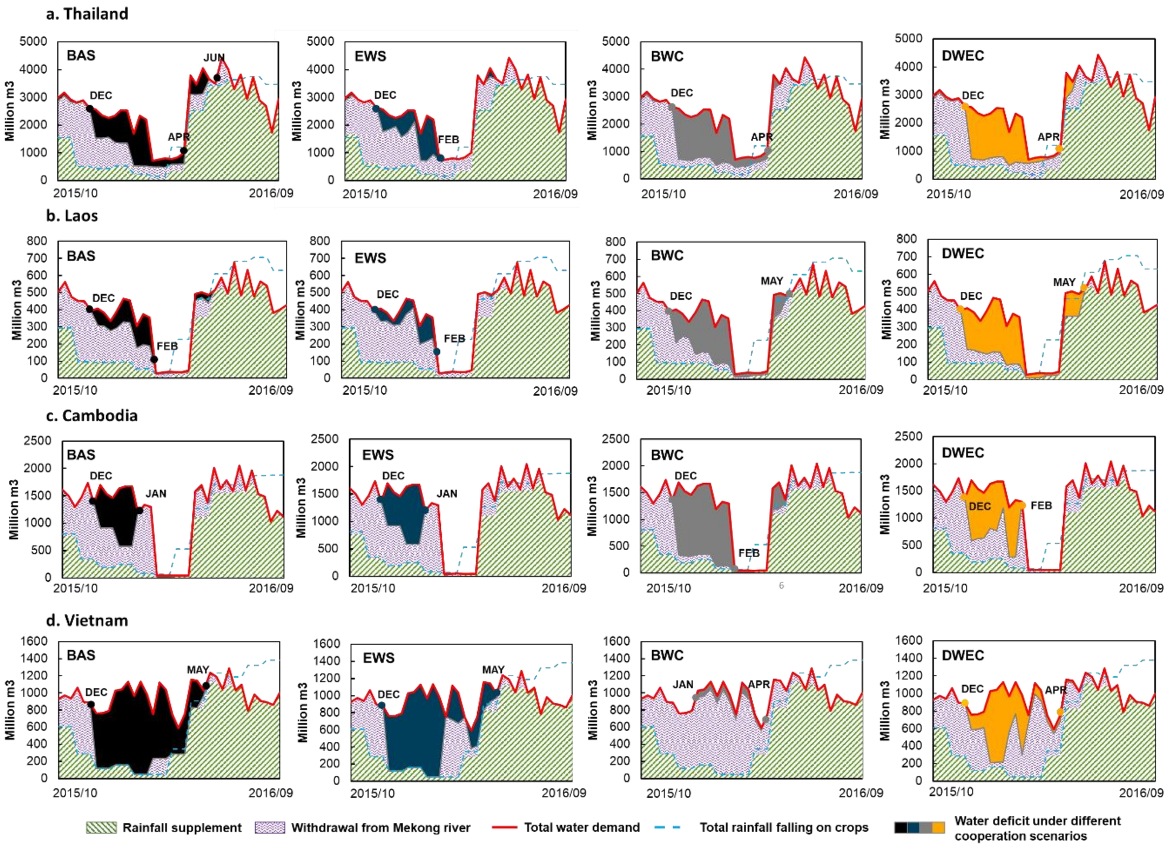

Supplementary Figure 2: Water deficit in various countries under different strategies: a. Thailand; b. Laos; c. Cambodia; d. Vietnam.

Note: The green shadow represents crops' water needs to be met by rainfall, and the purple shadow represents crops' water needs to be met by water withdrawal from the Mekong River. When the rainfall and water withdrawal still do not meet the water demand of crops (red line), there is a water deficit.

Acronyms: BAS, baseline with no cooperative action; EWS, Emergent Water Supplement; BWC, Basin-wide Water Cooperation; DWEC, Dual Water-Electricity Cooperation (BWC_CMin or BWC_WMax)

Description: Without cooperative action, downstream countries have different degrees of water shortage during dry periods, of which Vietnam is the most serious country. In emergent water supplement, as upstream China increases its outflow to downstream, the water deficit extent of downstream countries is alleviated. In basin-wide water cooperation, water also can be transferred among downstream countries to maximize the economic benefits of the entire basin. Thailand, Laos and Cambodia with lower unit water benefits transfer their partial water to Vietnam, which has higher unit water benefits, and thus, their water shortage becomes more serious. In dual water-electricity cooperation, water transfer is constrained by electricity trade capacity, and the amount of water transfers from Thailand, Laos and Cambodia is limited. Compared with the BWC scenario, Thailand, Laos and Cambodia's water deficit is improved in DWEC.

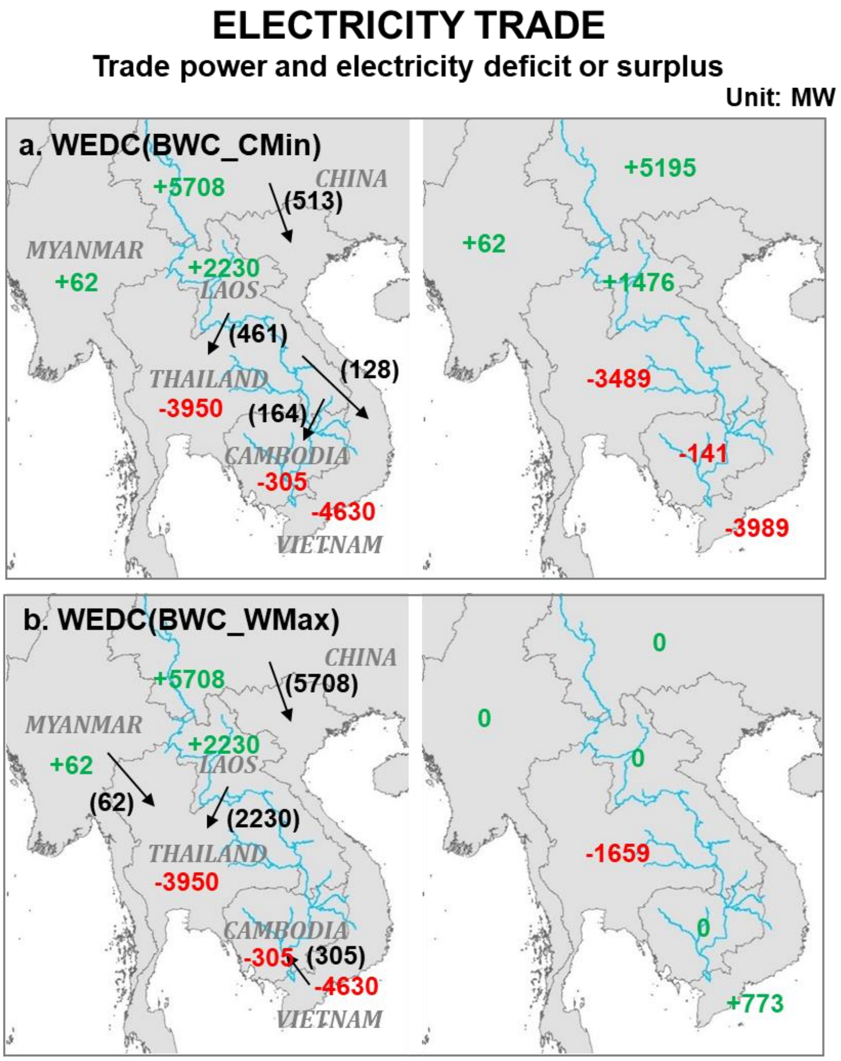

Supplementary Figure 3: Electricity deficit or surplus and trade power in dual water-electricity cooperation.

a. Dual Water-Electricity Cooperation developed on Basin-wide Water Cooperation and aimed for Cost Minimization (BWC_CMin);

b. Dual Water-Electricity Cooperation developed on Basin-wide Water Cooperation and aimed for Willingness Maximization (BWC_WMax);

Red number: electricity deficit; Green number: electricity surplus; values in (.) is the trade volume between two neighboring countries.

Supplementary Note 1: Data sources

The data involved in this study primarily include hydrometeorological data and socioeconomic data, and the Supplementary Table 1 summarizes the major data and their sources.

Supplementary Table 1: Data sources

Data		Source	Time scale
Hydro-meteorological data	Discharge	MRC (Mekong River Commission), available from: https://portal.mrcmekong.org/time-series/discharge	Long-series Daily
	Rainfall	TRMM, NASA (National Aeronautics and Space Administration). "Tropical rainfall measuring mission." Goddard Space Flight Center, available from: https://trmm.gsfc.nasa.gov/	Long-series Daily
	Temperature	ECMWF (European Centre for Medium-Range Weather Forecasts). "ECMWF public datasets: Global reanalysis ERA-20C.", available from: https://apps.ecmwf.int/datasets/data/era20c-daily/levtype=sfc/type=an/	Long-series Daily
	Radiation	Meteonorm, version 7.3, a software available from: https://meteonorm.com/	Horizontal year Daily
Socio-economic data	Crops yields, Prices, Areas	FAO (Food and Agriculture Organization). "Food and agriculture data: Crops and livestock products.", online database available from: https://www.fao.org/faostat/en/#data/QCL	Annual
	Crops calendar	FAO Irrigation and Drainage Papers[1-3] IRRI (International Rice Research Institute). "Rice knowledge bank, agronomy guides.", http://www.knowledgebank.irri.org/agronomy-guides	--
	Irrigation return flow	MRC, 2017, "Thematic report on the positive and negative impact of irrigation on the social, environment, and economic conditions of the Lower River Basin and Policy recommendations.", https://www.mrcmekong.org/assets/Publications/Council-Study/Council-study-Reports-Thematic/Impacts-of-Domestic-and-Industrial-Water-Use-28-Dec-2047.pdf	--
	Electricity availability; import and export;	IEA (International Energy Agency), IEA World Energy Balance Report, 2020 edition, available from: https://www.iea.org/data-and-statistics/data-product/world-energy-balances	Annual

	consumption per capital		
	Costs (CAPEX, OPEX)	References[4-6]	--

Supplementary Note 2: Calibration and validation of the SWAT model

The calibration and validation results of the hydrological model (SWAT) are shown in Figure S4. The calibration and validation period of the main hydrological stations are listed in Table S1, the period before 2005 can be regarded as a natural period with no significant human effect[7,8], and thus, the observed runoff can be directly used to calibrate and validate the model, whereas it is also limited by the lack of data. Two performance metrics are selected, i.e., Nash-Sutcliffe efficiency coefficient (NSE) and coefficient of determination (R^2). The value of NSE ranges from $-\infty$ to 1, if the value is closer to 1, it represents higher credibility and better performance of the model. R^2 represents the fitting degree of simulated value and observed value, the closer it is to 1, the better performance it indicates. The NSE of mainstream hydrological stations ranges from 0.85 to 0.97 in calibration periods and 0.80 to 0.97 in validation periods, which is considered to be satisfactory with the NSE > 0.6. Also, the coefficient of determination (R^2) ranges from 0.94 to 0.97 in calibration periods and 0.93 to 0.98 in validation periods. Both NSE and R^2 indicate the excellent performance of the SWAT model.

Supplementary Table 2: The calibration and validation period of the SWAT model

Location	Calibration period	Validation period
JH-Jinghong	1987 ~ 1995	1996 ~ 2003
CS-Chiang Sean	1962 ~ 1984	1985 ~ 2005
LP-Luang Prabang	1962 ~ 1984	1985 ~ 2005
VT-Vientiane	1962 ~ 1984	1985 ~ 2005
PA-Pakse	1962 ~ 1984	1985 ~ 2005
KR-Kratie	1962 ~ 1966	1967 ~ 1970

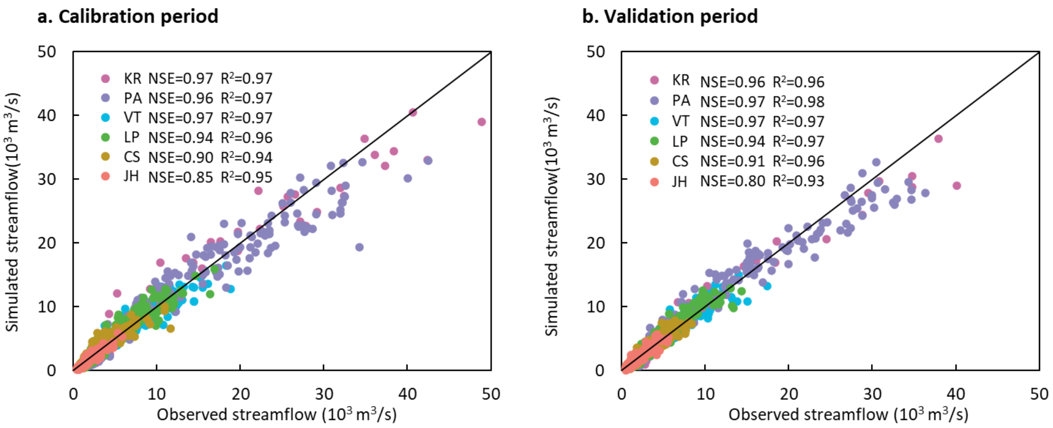

Supplementary Figure 4: Monthly runoff simulation results of the SWAT model. a. Calibration period; b. Validation period. Acronyms: JH-Jinghong; CS-Chiang Sean; LP-Luang Prabang; VT-Vientiane; PA-Pakse; KR-Kratie.

Supplementary Note 3: Willingness function.

We characterize the relationship between stakeholder's willingness and trade volume by triangle function, and the corresponding formula representations are as follows:

1. Type 1: Import appetite, water loss party. When the electricity trade compensates for the water loss, the stakeholder begins to be willing to cooperate with others, and the willingness increase as the electricity trade expands, until reaches the balance point at which the import electricity volume meets its deficit. The willingness can be described as follows:

$$WI_i = \frac{np_{e,i}}{np_{e,i} \times \Delta + B_i^w} \times T_i^{im} + \frac{B_i^w}{np_{e,i} \times \Delta + B_i^w}, T_i^{im} \in \left[-\frac{B_i^w}{np_{e,i}}, \Delta\right] \quad (1)$$

2. Type 2: Export appetite, water loss party. Similar to Type 1, the stakeholder begins to willing to cooperate with others as the water loss fully be compensated, until its abundant electricity is consumed by surrounding countries. The willingness can be described as follows:

$$WI_i = \frac{p_e}{np_{e,i} \times \Delta + B_i^w} \times T_i^{ex} + \frac{B_i^w}{np_{e,i} \times \Delta + B_i^w}, T_i^{ex} \in \left[-\frac{B_i^w}{np_{e,i}}, \Delta\right] \quad (2)$$

3. Type 3: Import appetite, water beneficiary. The stakeholder benefits from the water field and partial loss in the electricity trade can be tolerated. The loss in electricity means the trade development is not followed by the stakeholder's appetite, i.e. export or excessive import. The willingness function can be described as follows:

$$WI_i = \begin{cases} \frac{np_{e,i}}{np_{e,i} \times \Delta + B_i^w} \times T_i^{im} + \frac{B_i^w}{np_{e,i} \times \Delta + B_i^w}, & T_i^{im} \in [0, \frac{B_i^w}{np_{e,i}}] \\ -\frac{np_{e,i}}{B_i^w} \times T_i^{im} + \frac{np_{e,i} \times \Delta}{B_i^w} + 1, & T_i^{im} \in (\frac{B_i^w}{np_{e,i}}, \Delta] \\ -\frac{p_e}{np_{e,i} \times \Delta + B_i^w} \times T_i^{ex} + \frac{B_i^w}{np_{e,i} \times \Delta + B_i^w}, & T_i^{ex} \in [0, \frac{B_i^w}{np_{e,i}}] \end{cases} \quad (3)$$

4. Type 4: Export appetite, water beneficiary. Similar to Type 3, the stakeholder can undertake additional export or import within its water benefit. The willingness function can be described as follows:

$$WI_i = \begin{cases} \frac{np_{e,i}}{np_{e,i} \times \Delta + B_i^w} \times T_i^{ex} + \frac{B_i^w}{np_{e,i} \times \Delta + B_i^w}, & T_i^{ex} \in [0, \frac{B_i^w}{np_{e,i}}] \\ -\frac{np_{e,i}}{B_i^w} \times T_i^{ex} + \frac{np_{e,i} \times \Delta}{B_i^w} + 1, & T_i^{ex} \in (\frac{B_i^w}{np_{e,i}}, \Delta] \\ -\frac{p_e}{np_{e,i} \times \Delta + B_i^w} \times T_i^{im} + \frac{B_i^w}{np_{e,i} \times \Delta + B_i^w}, & T_i^{im} \in [0, \frac{B_i^w}{np_{e,i}}] \end{cases} \quad (4)$$

Where WI_i represents the willingness to participate in cooperation of stakeholder i; Δ refers to the difference between the demand and generation, when demand is greater than generation, means electricity deficit, Δ = demand - generation; also when demand is less than generation, means electricity surplus, Δ = generation - demand; B_i^w represents the benefits of stakeholder i brought by water cooperation, the value is negative when benefit loss; $np_{e,i}$ represents the net electricity price of stakeholder i, it equals the electricity economic price minus the costs that the stakeholder must afford; T_i^{im} and T_i^{ex} represents the import and export electricity trade volume by stakeholder$i$; $i, j \in N, N = 1,2,\cdots,6$.

Supplementary Note 4: Linkage between water and electricity module.

The linkage between the water and electricity module is taken by setting constraints. Considering the stakeholder's possible role in the water field (loss or gain) and electricity trade (import or export), linkages are described as follows:

From water module to electricity module:

1. Stakeholders with electricity import desire (i.e., demand is greater than generation) and water benefit loss in the dual cooperation, the loss in water cooperation is the lower limit for necessary electricity import.

$$-\frac{B_i^w}{np_{e,i}} \leq T_i^{im} \leq \Delta \tag{5}$$

$$0 \leq T_i^{ex} \leq 0, \text{i.e.} T_i^{ex} = 0 \tag{6}$$

2. Stakeholders with electricity export desire (i.e., demand is less than generation) and water benefit loss in the dual cooperation, its loss in water cooperation is the lower limit for necessary electricity export.

$$0 \leq T_i^{im} \leq 0, \text{i.e.} T_i^{im} = 0 \tag{7}$$

$$-\frac{B_i^w}{np_{e,i}} \leq T_i^{ex} \leq \Delta \tag{8}$$

3. Stakeholders with electricity import desire (i.e., demand is greater than generation) and water benefit gain in the dual cooperation, it can sustain additional import or export, and this part should not exceed its benefits gain in the water field.

$$0 \leq T_i^{im} \leq \frac{B_i^w}{np_{e,i}} + \Delta \tag{9}$$

$$0 \leq T_i^{ex} \leq \frac{B_i^w}{np_{e,i}} \tag{10}$$

4. Stakeholders with electricity export desire (i.e., demand is less than generation) and water benefit gain in the dual cooperation, it can sustain additional export or import, and this part should not exceed its benefits gain in the water field.

$$0 \leq T_i^{im} \leq \frac{B_i^w}{np_{e,i}} \tag{11}$$

$$0 \leq T_i^{ex} \leq \frac{B_i^w}{np_{e,i}} + \Delta \tag{12}$$

Where Δ refers to the difference between the demand and generation, when demand is greater than generation, means electricity deficit, Δ = demand - generation; also when demand is less than generation, means electricity surplus, Δ = generation - demand; B_i^w represents the benefits of stakeholder i brought by water cooperation,

the value is negative when benefit loss; $np_{e,i}$ represents the net electricity price of stakeholder i, it equals the electricity economic price minus the costs that the stakeholder must afford; T_i^{im} and T_i^{ex} represents the import and export electricity trade volume by stakeholder$i$; $i,j \in N, N = 1,2,\cdots,6$.

From the electricity module to the water module, Stakeholder's benefit loss in water would not be accepted when it exceeds the electricity trade compensation capacity.

1. Stakeholder with electricity import desire (i.e., demand is greater than generation), its electricity trade compensation capacity is determined by its own electricity import desire and electricity export provided by surrounding countries, as follows:

$$-\frac{B_i^w}{np_{e,i}} \leq \min\left(\Delta,\ \sum_{j\neq i} T_j^{im}\right) \tag{13}$$

2. Stakeholder with electricity export desire (i.e., demand is less than generation), its electricity trade compensation capacity is determined by its own electricity export desire and electricity import provided by surrounding countries, as follows:

$$-\frac{B_i^w}{np_{e,i}} \leq \min\left(\Delta,\ \sum_{j\neq i} T_j^{ex}\right) \tag{14}$$

References

1. Savva, A. P. & Frenken, K. *Crop water requirements and irrigation scheduling*. (Food and Agriculture Organization of the United Nations, Harare, 2002).
2. Allen, R. G., Pereira, L. S., Raes, D. & Smith, M. *Crop evapotranspiration: guidelines for computing crop water requirements*, FAO Irrigation and Drainage Paper 56 edn. (Food and Agriculture Organization of the United Nations, Rome, 1998).
3. Dastane, N. G. *Effective precipitation in irrigated agriculture*, FAO Irrigation and Drainage Paper 25 edn. (Food and Agriculture Organization of the United Nations, New York, 1974).
4. Chang, Y. & Li, Y. Power generation and cross-border grid planning for the integrated ASEAN electricity market: A dynamic linear programming model. *Energy Strateg. Rev.* **2**, 153-160 (2013).
5. Siala, K., Chowdhury, A. K., Dang, T. D. & Galelli, S. Solar energy and regional coordination as a feasible alternative to large hydropower in Southeast Asia. *Nat Commun*. **12**, (2021).
6. Chowdhury, A. F. M. K., Dang, T. D., Nguyen, H. T. T., Koh, R. & Galelli, S. The Greater Mekong's climate‐water‐energy nexus: how ENSO‐triggered regional droughts affect power supply and CO_2 Emissions. *Earth's Future*. **9**, (2021).

7. Li, Y. et al. Impact of climate change on the spatio-temporal characteristics of meteorological and hydrological drought over the Lancang-Mekong River basin. *Advances in water science*. **32**, 508-519 (2021).
8. MRC. Mekong Climate Change Adaptation Strategy and Action Plan. Vientiane, Lao PDR*: Mekong River Commission*; 2018.
